# Probing the deployment of peripheral visual attention during obstacle-crossing planning

**DOI:** 10.3389/fnhum.2022.1039201

**Published:** 2022-12-22

**Authors:** Raza N. Malik, Daniel S. Marigold, Mason Chow, Tania Lam

**Affiliations:** ^1^School of Kinesiology, University of British Columbia, Burnaby, BC, Canada; ^2^International Collaboration on Repair Discoveries, University of British Columbia, Vancouver, BC, Canada; ^3^Department of Biomedical Physiology and Kinesiology, Simon Fraser University, Burnaby, BC, Canada; ^4^Institute for Neuroscience and Neurotechnology, Simon Fraser University, Burnaby, BC, Canada

**Keywords:** visual attention, locomotion, movement planning, peripheral vision, obstacle avoidance

## Abstract

Gaze is directed to one location at a time, making peripheral visual input important for planning how to negotiate different terrain during walking. Whether and how the brain attends to this input is unclear. We developed a novel paradigm to probe the deployment of sustained covert visual attention by testing orientation discrimination of a Gabor patch at stepping and non-stepping locations during obstacle-crossing planning. Compared to remaining stationary, obstacle-crossing planning decreased visual performance (percent correct) and sensitivity (*d’*) at only the first of two stepping locations. Given the timing of the first and second steps before obstacle crossing relative to the Gabor patch presentation, the results suggest the brain uses peripheral vision to plan one step at a time during obstacle crossing, in contrast to how it uses central vision to plan two or more steps in advance. We propose that this protocol, along with multiple possible variations, presents a novel behavioral approach to identify the role of covert visual attention during obstacle-crossing planning and other goal-directed walking tasks.

## Introduction

Imagine walking on a trail through a forest; central vision (i.e., foveal fixation) identifies important stepping locations and objects in the walking path (Patla and Vickers, 1997; Marigold and Patla, 2007), while peripheral vision guides the lower limbs to the stepping locations or over hazardous objects as the fovea fixates on future areas of interest (Patla and Vickers, 1997; Mohagheghi et al., 2004). This simple scenario highlights the importance of peripheral vision in preparing human locomotion for expected gait modifications. To navigate everyday environments, the brain must deploy visual attention to relevant sensory stimuli to assist in formulating a movement plan and executing the desired action based on movement goals (Fecteau and Munoz, 2006; Gottlieb, 2007; Bisley and Goldberg, 2010). The allocation of visual attention can occur transiently (reflexively) in response to a sudden stimulus (Nakayama and Mackeben, 1989; Ling and Carrasco, 2006; Grubb et al., 2015) or in preparation for a movement (Baldauf et al., 2006; Rolfs and Carrasco, 2012; Rolfs et al., 2013; Stewart et al., 2019; Mahon et al., 2020), or it can be sustained (voluntary) based on behavioral objectives (Nakayama and Mackeben, 1989; Deubel and Schneider, 2003; Ling and Carrasco, 2006; Grubb et al., 2015). Numerous studies on the execution of skilled walking (e.g., obstacle crossing) in animals (Drew and Marigold, 2015) and humans (Patla, 1998; Niang and McFadyen, 2004; Marigold, 2008) have shed light on the role of central vision in the control of skilled walking. In contrast, behavioral experiments in humans that directly probe the role of peripheral visual attention during walking are lacking.

Attentional shifts can occur by directing gaze to a location of interest (overt attention) or with changes in visual attentional focus without eye movements, that is, using peripheral vision (covert attention). Reaching studies demonstrate a strong coupling between movement planning and covert selective attention. For example, preparing a reach enhances visual discrimination (Baldauf et al., 2006; Baldauf, 2011; Rolfs et al., 2013; Mahon et al., 2020) at covertly (peripherally) attended reach locations compared to non-reach locations. These enhancements in visual performance (percentage of correct responses) and visual sensitivity (*d’*) at the upcoming reach location occur when the test stimulus appears ~50–100 ms after the onset of the movement cue. This time course reflects a transient shift in visual attention towards the movement location (Müller and Rabbitt, 1989; Nakayama and Mackeben, 1989), indicating movement planning associated with a reflexive increase in visual attention at locations selected for action.

Before movement execution, attention may be inhibited at the movement location and re-directed towards other movement-relevant areas. This phenomenon occurs if there is ample time (>~300 ms) between the onset of a movement cue and the presentation of a test stimulus (Deubel and Schneider, 2003; Stewart et al., 2019). The re-direction of attention away from the movement location suggests a formulated movement plan is used to guide action execution with limited visual attention (Deubel and Schneider, 2003; Rolfs et al., 2013). The time course of ~300 ms reflects a sustained shift in attention (Müller and Rabbitt, 1989; Nakayama and Mackeben, 1989). Thus, transient and sustained attention may reflect the time course of how visual attention is deployed to prepare a goal-directed movement. Transient shifts in attention towards movement-relevant locations at short cue-test intervals suggest that these locations are rapidly selected for planning (Baldauf et al., 2006; Rolfs et al., 2013). Sustained shifts in attention away from movement locations, detected by longer cue-test intervals, imply that motor planning for the action is complete and that movement execution can proceed with limited visual attention (Deubel and Schneider, 2003).

Skilled walking tasks, such as obstacle crossing, are commonly encountered in daily life (Musselman and Yang, 2007). Obstacle locations, such as stairs and curbs, are often known and thus, people are well-trained in dealing with them. Because these obstacle locations are known in advance, a person can plan their gait adjustment; this contrasts with the appearance of unexpected obstructions that necessitate gait adjustments where there is little to no time to plan. Obstacle crossing, like many other walking tasks, relies on peripheral visual input to facilitate the planning and execution of the required gait adjustments (Marigold, 2008). Indeed, there is greater variation in foot placement and a higher incidence of obstacle contacts when peripheral vision from the lower visual field is restricted (Patla, 1998; Mohagheghi et al., 2004; Patla and Greig, 2006). But how is covert visual attention deployed to stepping locations in preparation for crossing an obstacle? Is covert attention selectively deployed to stepping locations over non-stepping locations to plan foot placement, or is it spread across the lower visual field with limited selectivity? In this study, we developed a novel behavioral approach to gain insight into the role of sustained covert visual attention (peripheral vision) in preparing foot placement during obstacle-crossing planning. We presented a cue at fixation indicating whether the participant should remain stationary or begin obstacle crossing and tested the modulation of visual performance and sensitivity between stepping vs. non-stepping locations. In this protocol, we used a long cue-test interval (300 ms), based on previous findings about the deployment of sustained visual attention, and predicted evidence for reduced sustained visual attention at the stepping locations. Reduced visual attention would be consistent with the notion that the steps were planned ahead of execution and manifest as decreased visual performance and sensitivity at stepping compared to non-stepping locations and the stationary baseline condition.

## Materials and Methods

### Participants

Twelve participants (six males and six females) with a mean age of 29 years (22–37), mean height of 173.9 cm (range 158–188), and mean weight of 71 kg (range 48–95) took part in this experiment. Eight participants were right-eye-dominant, and three participants were left-eye-dominant (data missing from one participant), as measured by the hole-in-card test. Participants did not have any self-reported neurological, musculoskeletal, visual, or attention disorders affecting mobility or cognition. Participants requiring visual correction wore contact lenses to improve gaze tracking with the eye-tracker. All participants provided informed, written consent and the University of British Columbia Clinical Research Ethics Board approved all experimental procedures.

### Setup

**Figure 1:**
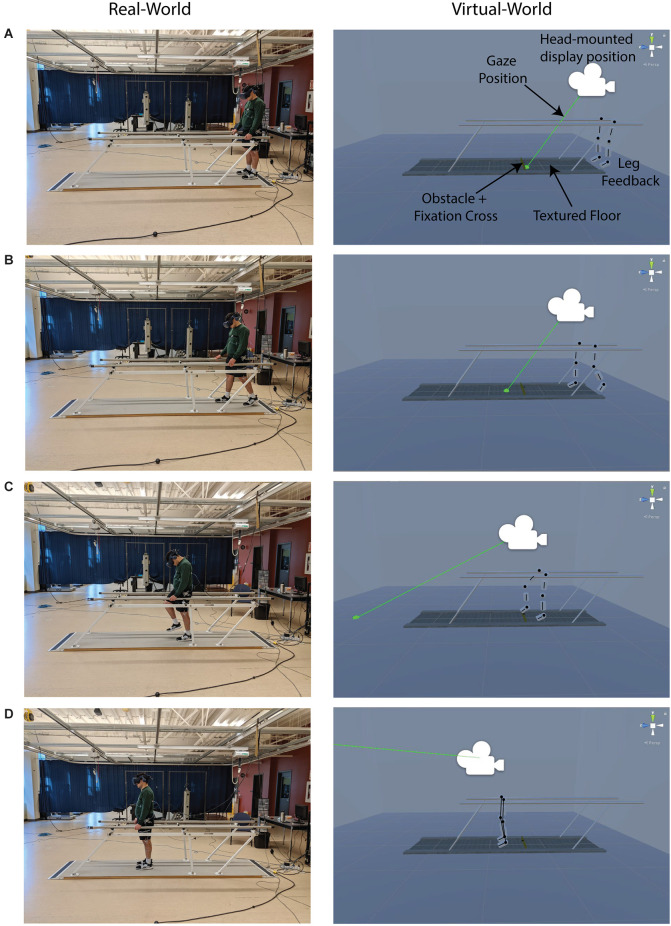
Real and virtual environments. Images of the real and virtual environment as a participant approached and stepped over a virtual obstacle. Participants received visual feedback of leg position throughout the experiment. In the virtual world, but not in the real world, the walkway had a textured floor surface and an obstacle with a fixation cross positioned two steps away from the participant. The four rows show the participant **(A)** at the starting position, **(B)** taking the first step, **(C)** stepping over the obstacle with the lead limb, and **(D)** at the end position. *The black markings over the legs are shown for visual clarity in this figure but were not present in the VR environment.

**Figure 2:**
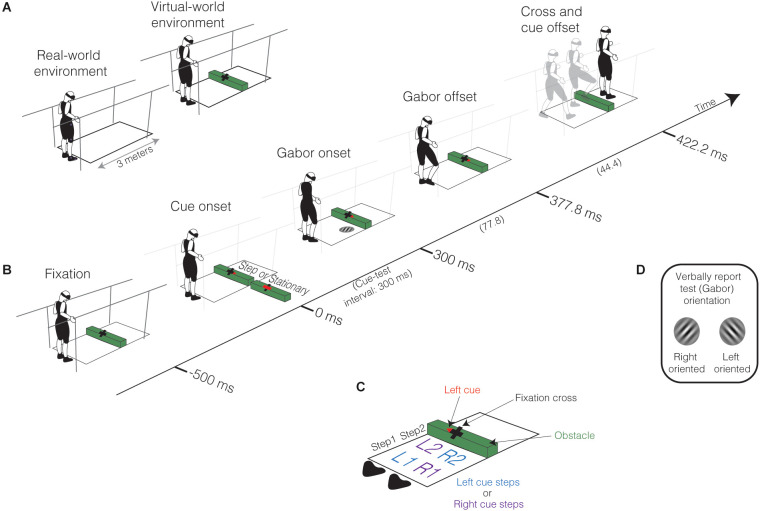
Experimental setup and procedures. **(A)** Participants stood on a 3-m platform between parallel bars while wearing a head-mounted display (HTC Vive Pro) embedded with a Pupil Labs eye-tracker. In the virtual world, but not in the real world, the floor surface had texture (not displayed here, see Figure 1), and a 5 cm obstacle placed two steps away from the participants’ starting position. Participants fixated on a cross placed on the obstacle to have them gaze two steps ahead. **(B)** Sequence of events in each trial. Fixation: Participants began by fixating on the cross for 500 ms. Cue onset: Following fixation, a cue presented at fixation prompts the preparation of obstacle crossing or to remain stationary. In this example, a rightward arrow cue is presented, indicating the participant should take their first step with the right leg as quickly as possible after cue onset. The cue could also be a leftward arrow, indicating the participant should begin stepping with their left leg first, or a horizontal bar indicating that they remain stationary. The cue consequently dictated the stepping and non-stepping locations along the walkway. Gabor Onset: The test stimulus (Gabor patch) was presented 300 ms after cue onset (cue-test interval of 300 ms). The Gabor patch appeared with equal probability at one of the four potential step locations (R1, L1, R2, L2, see **C**); in this example, the Gabor patch appears in R2. Gabor offset: The offset of the Gabor patch occurred 77.8 ms after its onset. Cross and cue offset: The offset of the cross and cue occurred 44.4 ms after the offset of the Gabor patch. On stationary trials, this is the end of the trial. On obstacle-crossing trials, participants initiated their first step within 1,000 ms after cue onset and approached and stepped over the obstacle. Participants also needed to verbally report the orientation of the Gabor patch as quickly as possible. **(C)** Potential stepping and Gabor patch locations determined by baseline step locations. There were two possible step locations in the first (step 1) and second (step 2) step, represented as the first: (1) and second (2) potential step locations for the Right (R) and Left (L) leg. For obstacle-crossing trials, the leftward arrow cue led to steps into positions L1 and R2 (L cue steps), and the rightward arrow cue led to steps into positions R1 and L2 (R cue steps), followed by stepping over the obstacle. **(D)** Orientation discrimination of the test stimulus (Gabor patch). Participants verbally reported the Gabor patch’s orientation relative to vertical (Right or Left) as quickly as possible following its presentation.

Participants wore a head-mounted display [HTC (New Taipei City, Taiwan) Vive Pro] with a built-in eye-tracker (Pupil Labs, Berlin, Germany) while standing at one end of a 3-m walkway between parallel bars (Figures 1, 2A). We projected a virtual reality (VR) environment into the head-mounted display created in Unity (Unity Technologies, San Francisco, USA). The VR environment mimicked the dimensions and characteristics of the real-world parallel bars and walkway (Figures 1, 2A). However, the VR walkway had texture and contained a virtual obstacle (5 cm high, 60 cm wide, 1 cm deep) placed two steps away from the participant (Figure 1). To enhance immersion, participants could view the position of their legs in the VR environment (Pastel et al., 2020). This was done by placing clusters of infrared emitting diodes (Optotrak, NDI, Waterloo, Canada) on the thorax and bilaterally on the greater trochanter, lateral femoral condyle, lateral malleolus, and head of the 5th metatarsal. We recorded this kinematic data at 100 Hz in Optotrak, which was then streamed into Unity *via* a custom-written routine in Vizard (World Viz Inc., Santa Barbara, USA) to generate the representation of the participant’s legs in the VR environment. To assess participants’ gaze in the VR environment, we recorded gaze data in real-time at 120 Hz in Pupil remote (a Pupil Lab’s software) and streamed it into Unity using a custom-written routine. We calibrated gaze data to Unity space prior to any data collection using the Pupil Lab’s hmd-eyes default 9-point calibration scene (Piumsomboon et al., 2017; Mutasim et al., 2020). Real-time kinematic and gaze data helped identify unusable trials due to errors in step onset and obstacle contacts or inappropriate eye movements (see “Data analysis” Section). We presented all visual stimuli in the VR environment on dual-OLED displays with a resolution of 2,880 × 1,600 (1,400 × 1,600 per eye, and 615 pixels per inch) with a display refresh rate of 90 Hz.

### Procedure

Our study required two laboratory visits. In the first visit, we familiarized participants with the obstacle-crossing task in the VR environment. We then conducted threshold testing to determine their 79% orientation discrimination threshold to the test stimulus, which was a Gabor patch (a sinusoidal wave pattern convolved with a Gaussian envelope, see Figure 2D). In the second visit, we conducted the experimental testing of perceptual performance in Gabor orientation discrimination during the obstacle-crossing task.

#### Obstacle-crossing task

Figure 2B shows the sequence of events in a single trial. To begin each trial, participants fixated on a central cross located on the obstacle while standing at their start position (*Fixation*, Figure 2B). Subsequently, participants received a verbal cue to prepare for the upcoming trial. After 500 ms, a cue appeared (*Cue onset*, Figure 2B) to direct the participant to either begin obstacle crossing (rightward or leftward arrow) or remain stationary (horizontal bar). On obstacle-crossing trials, the arrow indicated the leg the participant should start walking with. For example, a rightward arrow cue meant the participant should take their first step with their right leg; the cue consequently dictated the stepping and non-stepping locations. For rightward arrows, participants stepped into R1 and L2; for leftward arrows, L1 and R2 (Figure 2C). The visual angle of the arrow cue relative to the center of the fixation cross slightly varied between participants due to differences in height. On average, the arrow cue appeared 1.23° (range: 1.15°–1.37°) to the right or left of the fixation cross. Stationary trials indicated baseline performance when participants were not required to approach and step over the obstacle. Notably, the cue did not predict the spatial location of the Gabor patch and only indicated movement initiation or remaining stationary (Baldauf et al., 2006; Rolfs et al., 2013); we explicitly made participants aware of this before the start of the experiment.

After a cue-test interval of 300 ms (*Test onset*, Figure 2B), we presented the Gabor patch, which we oriented to either the left or right (with respect to vertical), for 77.8 ms (*Test offset*, Figure 2B). The Gabor patch, which had a contrast of 18%, a spatial frequency of six cycles/pixels, and a diameter of 9.5 cm, had an equal probability of appearing at one of four locations (*R1, L1, R2, L2*; Figure 2C). The cross and movement cue disappeared 44.4 ms after removing the Gabor patch (*Cross and cue offset*, Figure 2B). In stationary trials, cross and cue offset indicated the end of the trial. In walking trials, participants continued the obstacle-crossing action, taking two steps and stepping over the virtual obstacle with both the lead (first leg over the obstacle) and trail (second leg over the obstacle) legs for a total of four steps. We instructed participants to verbally report the orientation of the Gabor patch (Figure 2D) as quickly as possible while they initiated obstacle crossing (or remained stationary). We recorded the verbal response after each trial as a dichotomous variable indicating if the participant correctly or incorrectly identified the orientation of the Gabor patch. We did not provide performance feedback regarding test orientation, as discrimination accuracy is influenced by feedback (Siedlecka et al., 2020). The time it took to report the orientation of the Gabor patch was not recorded due to methodological limitations. However, previous research has confirmed that non-speeded responses show the expected changes in perceptual performance in response to a movement cue (Baldauf et al., 2006; Rolfs et al., 2013) and a spatial cue (Handy et al., 2001).

During obstacle crossing and stationary trials, visibility of the fixation cross indicated participants needed to maintain gaze fixation on the cross within 2°, ensuring the use of covert and not overt mechanisms for discriminating the Gabor patch. During obstacle-crossing trials, we instructed participants to initiate their step with the correct foot as quickly as possible upon cue presentation. Step onset needed to occur between 377.8 ms (Gabor patch offset) and 1,000 ms after cue-onset. Participants also needed to step over (clear) the virtual obstacle, but we did not give them any specific instructions to clear the obstacle with any level of precision. If the participant violated gaze fixation, step onset, or obstacle contact criteria, they were notified verbally, and the trial was discarded. Discarded trials were re-collected at the end of the block.

#### Familiarization (visit 1)

Participants started by walking along the walkway to confirm that their walking speed and leg movements in the VR environment felt natural and coincident with their real-world actions. Once comfortable, we determined their starting position relative to the obstacle. We adjusted each participant’s start position to be two steps away from the obstacle to account for differences in stride length. We chose this start position because previous walking studies show participants generally gaze at an obstacle two steps away from it (Patla and Vickers, 1997). Once the participant displayed consistency in their obstacle-crossing performance in the virtual environment, we collected 10 walking trials (five with the right movement cue and five with the left movement cue, presented in random order). We used these baseline trials to determine the participant’s average stepping locations for the right and left cues as they approached and stepped over the obstacle. These stepping locations defined the four spatial locations in which the Gabor patch could be placed on the walkway, depending on which leg stepped first *(R1, L1, R2, L2*; Figure 2C) during the obstacle-crossing trials. We made participants aware of their stepping locations and told them to maintain a similar walking pattern throughout the experiment to the best of their abilities. The average visual angles across all participants for R1, L1, R2, and L2 relative to the center of the fixation cross were 24.0° (range: 21.2°–27.5°), 23.7° (range: 21.3°–26.8°), 8.5° (range: 6.3°–10.9°), and 8.1° (range: 5.5°–11.1°), respectively.

#### Threshold testing (visit 1)

We conducted threshold testing to obtain the 79% orientation discrimination threshold of the Gabor patch at each location during obstacle-crossing trials. We used an interleaved 3-down/1-up staircase procedure to conduct threshold testing at each location simultaneously (Levitt, 1971; Leek, 2001). The step size of the staircase was 0.3°, and the starting orientation was 6° from vertical (Rolfs et al., 2013). The staircase procedure terminated after the sixth reversal (Levitt, 1971), and we calculated the 79% orientation discrimination threshold by averaging the last four reversal values (Amitay et al., 2006). We conducted the staircase procedure in the same environment and conditions as the obstacle-crossing task, but without the stationary trials. The direction of the cue and the location and orientation of the Gabor patch were all randomized. The 79% orientation discrimination threshold determined at each stepping location dictated the degree of rotation from the vertical of the Gabor patch at each location during the testing session. For example, a 79% orientation discrimination threshold of 2° from vertical in step R2, meant the orientation of the Gabor patch at position R2 during the testing session was always 2° to the left or right from vertical. We determined a threshold for each stepping location to control for eccentricity effects on perceptual performance (Carrasco et al., 2001; Carrasco, 2011). On average participants completed 354 trials (range: 200–443 trials) during threshold testing.

#### Experimental testing (visit 2)

During the testing session, participants completed four blocks of 72 trials. This included 48 walking trials and 24 stationary trials in each block, resulting in 192 walking trials (divided equally between right- and left-cued trials) and 96 stationary trials (288 trials in total). We presented the Gabor patch at each location (R1, L1, R2, L2) 12 times, six times with a right cue and six times with a left cue for walking trials in a single block, evenly distributing the number of trials initiated with the right or left leg. For stationary trials in a single block, we presented the Gabor patch at each location six times. Participants took rest breaks as needed and completed one block at a time with a 10–15 min break between blocks.

Following the completion of the testing session, we characterized the overall weighted subjective workload of the task and presence in the VR environment using the NASA Task Load Index (TLX; Hart and Staveland, 1988) and presence questionnaire (Witmer et al., 2005), respectively. The NASA TLX weighs the overall workload by six factors: mental demand, physical demand, temporal demand, performance, effort, and frustration. Higher scores indicate a more significant overall workload, with a maximum score of 100 (Grier, 2015). The presence questionnaire inquired about four factors: involvement, adaptation/immersion, interface quality, and sensory fidelity. Higher scores indicate a greater presence in the VR environment, with a maximum score of 154.

### Data analysis

We exported gaze and kinematic data from Unity and analyzed it using custom-written routines in MATLAB (MathWorks, Natick, MA).

#### Gaze fixation

We calculated the visual angle in the horizontal and the vertical planes during the fixation to cross offset period (Figure 2B) using two-dimensional gaze position to confirm offline whether participants-maintained fixation on the central cross. This ensured discrimination judgments of the Gabor patch relied on peripheral vision and covert attention mechanisms. We discarded trials if the visual angle deviated by more than 2° in any direction from the initial fixation position in the presence of the fixation cross. We excluded 0.1% of trials due to unsatisfactory gaze fixation.

#### Obstacle-crossing behavior and step performance

**Figure 3:**
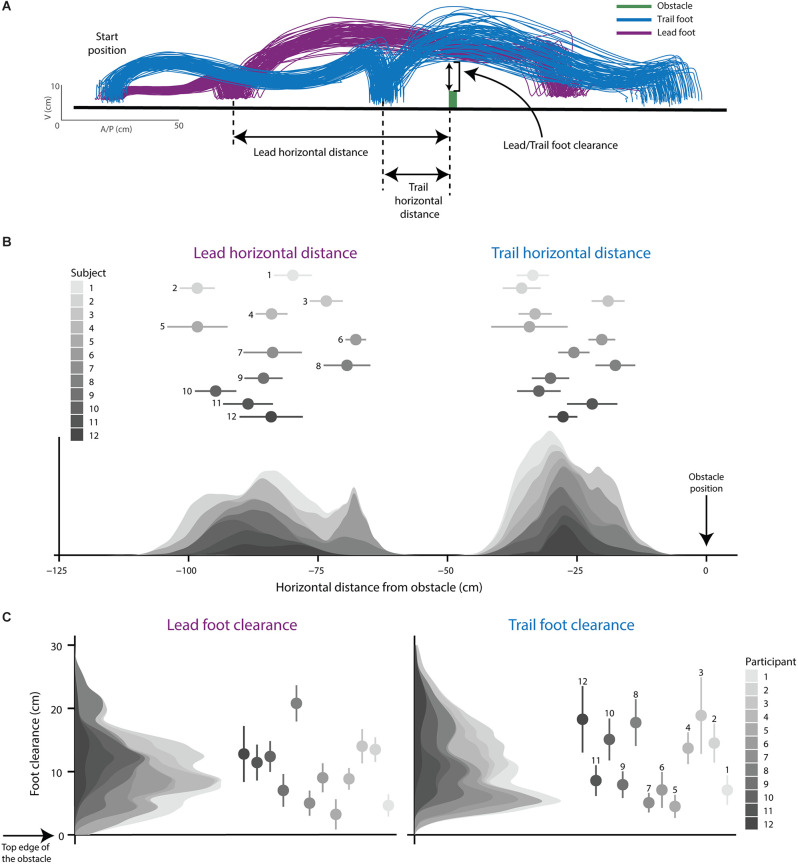
Obstacle-crossing behavior. **(A)** Foot trajectories of the lead (the first leg to cross obstacle) and trail (the second leg to cross obstacle) limb from a representative participant as they approached and stepped over the obstacle. Lead and trail horizontal distance are shown and defined as the horizontal distance between the obstacle and the foot in the step before crossing the obstacle. Lead and trail foot clearance is depicted and defined as the vertical distance between the obstacle and the foot at the point of crossing the obstacle. **(B)** Stacked density plots for lead and trail horizontal distance for each participant. **(C)** Stacked density plots for lead and trail foot clearance for each participant. Dots and error bars represent the mean and 95% CIs for each participant.

The placement and clearance of the first (lead) and second (trail) limb over an obstacle is important for avoiding obstacle contact (Patla and Greig, 2006). Thus, we characterized the walking pattern during obstacle crossing by the horizontal distance and vertical clearance of the first (lead) and second (trail) foot relative to the obstacle. We used the marker placed on the head of the 5th metatarsal to define the foot, and defined *Lead and trail horizontal distance* as the distance between this foot marker of the lead or trail limb and the front edge of the obstacle in the step before obstacle crossing (Figure 3A). We defined *lead and trail foot clearance* over the obstacle as the vertical distance between the top edge of the obstacle and the lead or trail foot marker at the time of obstacle crossing (Figure 3A).

During obstacle-crossing trials, we used *step onset* to define the planning (cue onset to step onset) and execution (after step onset) stages of the movement. We quantified step onset by the first deviation of the foot marker in either the forward or upward direction of at least 0.5 cm relative to the onset of the cue. We discarded the trial if step onset occurred before the offset of the Gabor patch (<377.9 ms) or if it was >1,000 ms following cue onset. These criteria ensured that we only included trials where discrimination judgments occurred during movement planning.

We calculated *foot placement* relative to the test location to ensure participants’ stepping locations coincided with Gabor locations. We did this to confirm discrimination judgments occurred at potential stepping locations. We calculated *foot placement* as the vector distance between foot position at midstance and Gabor patch location on the walkway for each step. We did not require participants to achieve a minimum step accuracy; thus, we did not exclude trials based on foot placement if the step sequence was correct.

In total, we discarded 2.84% of the kinematic data; 1.42% of trials because the participant started the movement too fast (0.07%), too slow (0.59%), or from unusable kinematic data (0.76%).

#### Gabor orientation discrimination responses

We grouped all verbal responses (correct/incorrect) according to whether the Gabor patch appeared at a stepping or non-stepping location and if the Gabor patch appeared in the first or second step. The direction of the cue determined the relevance (stepping or non-stepping) of a location such that for right-cued trials, the stepping locations are positions R1 and L2 on the walkway, and non-stepping locations are L1 and R2 (*Right cue steps*, Figure 2C), and *vice versa* for left cue trials (*Left cue steps*, Figure 2C). For the obstacle-crossing trials, we binned responses into the four possible combinations of relevance and step. For stationary trials, we binned responses only by step (step1 or step2) because relevance (stepping or non-stepping locations) is not applicable for these trials.

We then characterized the verbal responses at each location by using signal detection theory to calculate visual performance (percent correct), visual sensitivity (*d’*), and response bias (β; Stanislaw and Todorov, 1999; Pallier, 2002; Macmillan and Creelman, 2004). We calculated these measures in R with the dprime function, which uses the formulas (see below) from Pallier (2002) in the package psycho (Makowski, 2018). To test whether the changes in visual performance resulted from modifications in visual processing or from changes in response bias (i.e., a general bias to respond “right” or “left”), we calculated *d’* and β, in addition to percent correct (Macmillan and Kaplan, 1985; Pallier, 2002). We first categorized each verbal response as a hit, miss, correct rejection, or false alarm. We arbitrarily classified a *hit* as a correct response to a right-oriented Gabor patch, a *miss* as an incorrect response to a right-oriented Gabor patch, a *correct rejection* as a correct response to a left-oriented Gabor patch, and a *false alarm* as an incorrect response to a left-oriented Gabor patch. We then used these variables to calculate the percent correct (Pallier, 2002; Macmillan and Creelman, 2004):


(1) $$\%correct=\frac{\left(hits+correctrejections\right)}{\left(hits+misses+correctrejections+falsealarms\right)}\times 100$$

Percent correct reflects overall discrimination performance and is a function of both visual sensitivity for orientation discrimination and response bias (Macmillan and Kaplan, 1985; Pallier, 2002). To calculate sensitivity and bias, we first calculated the hit rate and false alarm rate as (Stanislaw and Todorov, 1999; Pallier, 2002; Macmillan and Creelman, 2004):


(2) $$hitrate=\frac{hits}{\left(hits+misses\right)}$$

(3) $$falsealarmrate=\frac{falsealarms}{falsealarm+correctrejections}$$

We used the log-linear rule—add 0.5 to the numerator and 1 to the denominator—to calculate each participant’s hit and false alarm rates to account for perfect performance resulting in an extreme hit and false alarm rates of 1 and 0 (Hautus, 1995). We then calculated visual sensitivity (*d’*) and response bias (β) as (Stanislaw and Todorov, 1999; Pallier, 2002; Macmillan and Creelman, 2004).


(4) $$d^{'}=Z\left(hitrate\right)-Z\left(FArate\right)$$

(5) $$\beta =\exp\left(\frac{-Z\left(hitrate\right)\times Z\left(hitrate\right)}{2}+\frac{Z\left(FArate\right)\times Z\left(FArate\right)}{2}\right)$$

For *d’*, a value of 0 represents chance performance, with higher values indicating increased discrimination sensitivity. For β, a score of 1 reflects an unbiased participant, scores closer to 0 reflect a right-biased participant, and scores greater than 1 reflect a left-biased participant.

We calculated changes (Δ) in percent correct, *d’* and β by subtracting scores during the stationary trials from those during the obstacle-crossing trials for a given step. For example, we subtracted scores at the stepping and non-stepping locations in step1 and step2 by stationary scores in step1 and step2, respectively. Negative values indicate scores were higher during the stationary condition.

### Statistical analysis

We used RStudio Version 1.3.1093 for all statistical analyses, and an α of 0.05 indicated significance for all statistical tests. We used descriptive statistics, including means and standard deviation, frequency, and percentages, as appropriate, to summarize participant characteristics, gaze fixation (visual angle), obstacle-crossing behavior (vertical and horizontal foot position relative to the obstacle), step performance (step onset and step placement), and orientation discrimination (percent correct, d’, β). We used dot plots, histograms, and density plots to visualize the data. The outcome variables entered into the statistical models below included the continuous variables Δ percent correct, Δ d’, Δ β, step onset, and foot placement.

We fitted linear mixed-effects models (LMEs) to enhance understanding of the relationship between outcomes and predictor variables while accounting for repeated observations within a participant. Predictor variables (i.e., fixed effects) were relevance (stepping, non-stepping), and step (step1, step2), and the referents were stepping and step1, respectively. To rule out any practice or eye dominance effects, we confirmed that these variables were not significantly associated with visual performance, and therefore were not included in the LMEs. For all LMEs, we accounted for the correlation in the data due to participants by using a random intercept model that controlled for the random effect of participants. We modeled the LMEs in R (R Core Team, 2020) using the lme4 package (Bates et al., 2015). We generated and visualized tables of estimates, confidence intervals, p values, and random effects using the sjPlot package (Lüdecke, 2020). We assessed model fit using information criteria [Akaike Information Criteria (AIC)], where smaller values represent a better fitting model (Hoffman, 2015). The Wald test and AIC identified the best-fitting model, and we used Maximum Likelihood to estimate all models.

During obstacle-crossing planning, we identified the effect of relevance and step on percent correct, *d’*, β, step onset, and foot placement by examining the association between relevance and step with Δ percent correct, Δ *d’*, Δ β, step onset, and foot placement. We did this by generating a model that included the fixed effects of relevance (stepping/non-stepping) and step (step 1/step 2) and the random effect of participants. We investigated the interaction between relevance and step to assess if step modified the relationship between relevance and the outcome variable. We only included the two-way interaction in the model if it was statistically significant and improved the overall fit of the final model. We used Cohen’s *f*^2^ to determine the effect size of the variance explained by each model (marginal R^2^). We also used Cohen’s *f*^2^ to calculate the size of a given fixed effect by comparing the variance explained in a model with that given fixed effect with a model without the given fixed effect (Lorah, 2018). For Cohens *f*^2^, effect sizes of 0.02, 0.15, and 0.35 were considered small, medium, and large, respectively (Cohen, 1992).

We further evaluated meaningful interactions with pairwise comparisons using the emmeans package (Lenth, 2020). We were interested in four comparisons from the two-way interaction: the first stepping location compared to: (1) the second stepping location; (2) the first non-stepping location; (3) the second non-stepping; and (4) the second stepping location compared to the second non-stepping location. We, therefore, used an adjusted p-value of 0.013 based on a Bonferroni correction for four comparisons. We used Cohen’s *d* to determine the effect size of estimated mean group differences for the pairwise comparisons. For Cohens *d*, effect sizes of 0.2, 0.5, and 0.8 were considered small, medium, and large, respectively (Cohen, 1992).

We assessed estimates of the change scores at each stepping and non-stepping location and their 95% confidence intervals to test differences between obstacle-crossing planning and remaining stationary. For the change scores, a score of zero represents the same score as the stationary trials. Thus, change scores with confidence intervals that did not cross zero were considered statistically different (*p* < 0.05) than scores from the stationary condition. We used Cohen’s *d* to determine the effect size of mean change scores compared to zero.

We conducted model diagnostics for the LMEs using the DHARMa (Hartig, 2020) package on each outcome’s final model. The QQ (quantile-quantile) plot of residuals and the plot of residuals against predicted values tested the assumptions of normality and homogeneity of variance, respectively. The outcome variables did not require data transformation.

## Results

All participants indicated high presence in the VR environment, shown by a mean presence questionnaire score of 124.2 (range 110–144.5; Witmer and Singer, 1998; Costa et al., 2018). Participants also showed a high workload to complete this task indicated by a mean NASA TLX score across participants of 59.4 (range 38.7–77.3; Grier, 2015). The 79% orientation discrimination thresholds for each potential stepping location were 4.3° (range 1.60–7.60) for L1, 4.0° (range 1.60–7.30) for R1, 3.6° (range 2.05–5.65) for L2, and 3.2° (range 1.50–5.85) for R2.

### Gaze fixation

The mean horizontal and vertical visual angles during the fixation to cross offset period were 6.54 × 10^−6^ degrees (SD 9.54 × 10^−4^) and −2.1 × 10^−3^ degrees (SD 2.5 × 10^−3^), respectively. These visual angles show that the gaze was fixated on the cross when the Gabor patch was presented, confirming visual discriminations of the Gabor patch occurred using covert attention.

### Obstacle-crossing behavior and step performance

The foot trajectory of a representative participant’s lead and trail limbs indicates real-world obstacle-crossing behavior in the virtual reality world (Figure 3A). Figures 3B,C show the distribution of foot placement and foot clearance, respectively, using stacked density plots where each shading gradient represents individual data from each participant. The mean lead and trail horizontal distance from the obstacle were 84.0 cm (SD 10.8) and 27.6 cm (SD 7.5; Figure 3B). The mean lead and trail foot clearance over the obstacle were 10.0 cm (SD 5.4) and 11.3 cm (SD 6.1; Figure 3C).

**Figure 4:**
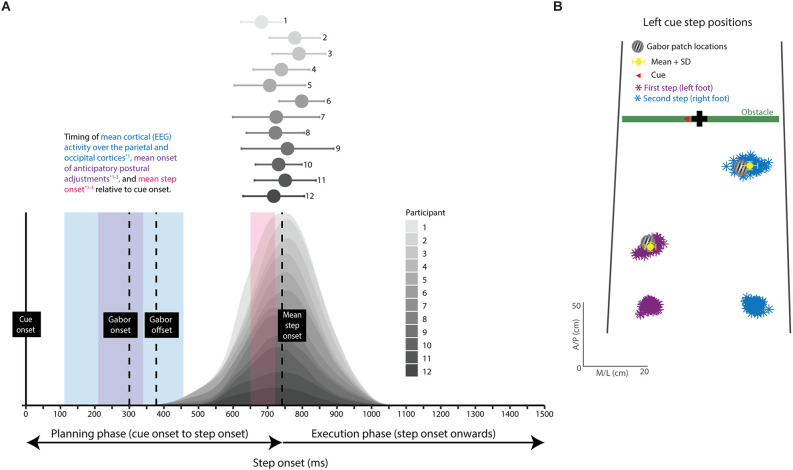
Step performance (step onset and foot placement). **(A)** Stacked density plots of step onset time for each participant; dots and error bars represent the mean and 95% CI. Vertical dotted lines represent important time points from our study. The vertical lines (left to right) represent Gabor patch onset, Gabor patch offset, and mean step onset relative to cue onset. The purple, blue, and pink shaded regions represent the timings for indicators of movement planning extracted from the literature. Changes in the center of pressure (purple), referred to as anticipatory postural adjustments, and cortical (EEG) activity (blue) over the parietal and occipital lobes occur shortly after the onset of a movement cue (~100–450 ms) and indicate movement planning. Step onset (pink) occurs later (~650–710 ms) and represents the end of the planning phase and the beginning of the execution phase. The step onset times in our study were similar to step onset times in previous studies (right most vertical line vs. pink shaded region), making it likely that cortical activity and anticipatory postural adjustments in our participants began shortly after cue onset. The overlap between the indicators of movement planning and the onset and offset of the Gabor patch show that our participants likely visually discriminated the Gabor patch during the planning phase of the movement. **(B)** Foot placement relative to test stimulus (Gabor patch) location. The starting position and foot placement for every left cue trial from a representative participant. The Gabor patch depicts the test stimulus location. The colored asterisks represent the left (purple) and right (blue) steps, and the yellow dot and error bars represent the mean and standard deviation (SD). EEG: electroencephalography. *1: Braquet et al. (2020). *2: Uemura et al. (2013a). *3: Uemura et al. (2013b).

Step onset times imply visual discriminations occurred during the planning phase of obstacle crossing. The distribution of step onset times for each participant are shown in Figure 4A using stacked density plots, where each shading gradient represents individual data from each participant. During the obstacle-crossing trials, participants initiated their movements quickly (<1,000 ms) and after the Gabor patch offset. The average step onset was 741 ms (SD 105 ms), which occurred well after the offset of the Gabor at 377.8 ms (Figure 4A). Moreover, step onset times were not associated with relevance or step (Table 1). Based on data from others (Uemura et al., 2013a,b; Braquet et al., 2020), providing biomechanical and neurophysiological indicators of movement planning (see figure legend), visual processing in our study likely occurred during movement planning (Figure 4A; see also “Discussion” Section).

**Table 1:** Linear mixed effect models (LMEs) fitted to estimate the relationship between the predictor variables with step onset and vector distance of the first and second steps.

	**Step onset**				**Vector distance (first step)**				**Vector distance (second step)**			
***Predictors***	***Estimates***	***95% CI***	***p***	***f2***	***Estimates***	***95% CI***	***p***	***f2***	***Estimates***	***95% CI***	***p***	***f2***
(Intercept)	0.740	0.719–0.762	**<0.001**		5.863	4.668–7.057	**<0.001**		7.085	5.248–8.922	**<0.001**	
Relevance [non-stepping]	−0.001	−0.009–0.007	0.8770	0.000	0.086	−0.157–0.329	0.488	0.001	0.076	−0.152–0.305	0.512	4.61 × 10^–5^
Step [Step 2]	0.001	−0.007–0.009	0.7567	0.000	0.233	−0.010–0.476	0.060	7.76 × 10–^5^	−0.060	−0.288–0.168	0.605	0.000
**Random Effects**												
Residual variance	2.0 × 10^–4^				0.184				0.163			
Random intercept variance	0.001				4.320				10.419			
ICC	0.866				0.959				0.985			
Observations	48				48				48			
Marginal R^2^/Conditional R^2^	0.000/0.866			0.000	0.003/0.959			0.001	0.000/0.985			4.61 × 10^–5^

Referent: Stepping, step 1; ICC, Intraclass Correlation Coefficient; f2, Cohen’s f2, indicating effect size. f2 for the model (bottom row) is calculated from the Marginal R2. Significant *p* values (i.e., <0.05) are bolded.

Foot placement relative to the Gabor location for the first and second stepping locations confirmed visual discriminations took place at stepping and non-stepping locations (Figure 4B). An overall vector distance of 6.0 cm (SD 3.6) and 7.1 cm (SD 4.7) between foot position and Gabor location in the first (R1 and L1) and second steps (R2 and L2), respectively, indicate participants stepped very close to the Gabor patch locations. Foot placement of the first or second step lacked associations with relevance or step (Table 1).

### Gabor orientation discrimination responses

**Figure 5:**
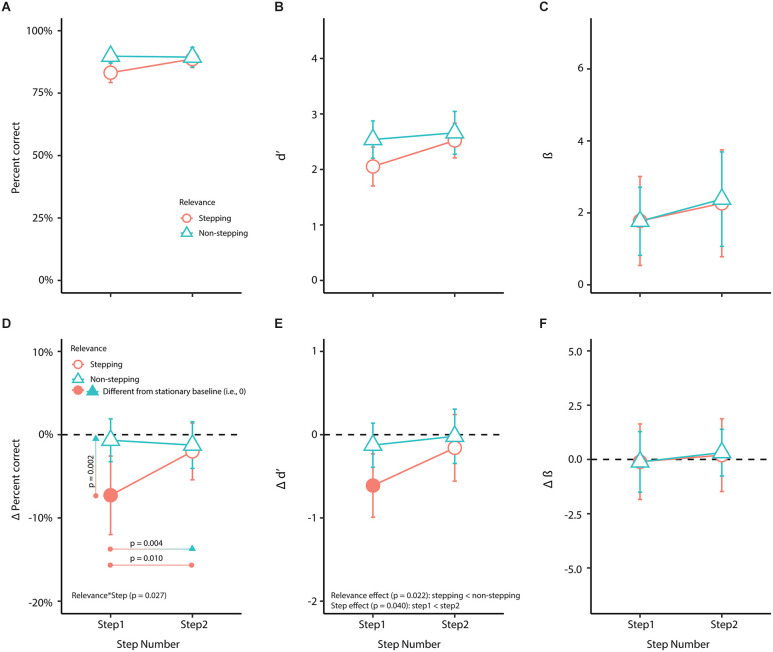
Orientation discrimination. This figure shows raw and change (Δ) scores (obstacle-crossing scores minus stationary scores) factored by relevance and step for **(A)** percent correct (visual performance); **(B)** d’ (visual sensitivity); **(C)** β (response bias); **(D)** Δ percent correct (Δ visual performance); **(E)** Δ d’ (Δ visual sensitivity); and **(F)** Δ β (Δ response bias). In the bottom panel **(D–F)**, the horizontal dashed line represents scores from the stationary baseline. Filled symbols represent values that were statistically different (*p* < 0.05) from the stationary baseline, determined by whether the 95% confidence intervals (error bars) crossed zero. We used linear mixed-effects models for statistical analyses on the Δ scores (Table 2). Pairwise comparison *p* values in **(D)** were evaluated at a Bonferroni corrected alpha value of 0.013 (see “Material and methods” Section).

The raw scores for visual performance, sensitivity, and response bias are plotted in Figure 5. Raw scores were plotted for descriptive purposes (Figures 5A–C), but statistical analyses were conducted on the change scores (Figures 5D–F; see “Methods” Section).

Obstacle-crossing planning decreased visual performance and sensitivity at the first stepping location compared to remaining stationary. The estimates, confidence intervals, and p-values for visual performance and sensitivity at the first stepping location are shown by the intercepts in Table 2; as the first stepping location is the referent in the LMEs. The intercepts, which compare the referent to zero (i.e., the stationary scores), indicate that percent correct and *d’* at the first stepping location during obstacle-crossing planning decrease by 7.3% (*p* = < 0.001, *d* = 1.39) and 0.52 units (*p* = < 0.001, *d* = 1.11), respectively, compared to remaining stationary. Only the 95% confidence intervals for Δ percent correct and Δ *d’* at the first stepping location do not cross zero, indicating they significantly differed from percent correct and *d’* when remaining stationary (Figures 5D–E, *filled dots*). Confidence intervals for Δ percent correct and Δ *d’* at the second and non-stepping locations cross zero, indicating their scores are similar to stationary scores (Figures 5D–E, *unfilled dots*). Obstacle-crossing planning did not alter Δ response bias at any stepping or non-stepping locations compared to remaining stationary (Figure 5F, *unfilled dots*).

**Table 2:** Linear mixed effect models (LMEs) fitted to estimate the relationship between the predictor variables and Δ percent correct (visual performance), Δ *d’* (visual sensitivity), and Δ beta (response bias).

	**△ % Correct (visual performance)**				**△ *d’* (visual sensitivity)**				**△ Beta (response bias)**			
***Predictors***	***Estimates***	***95% CI***	***p***	***f2***	***Estimates***	***95% CI***	***p***	***f2***	***Estimates***	***95% CI***	***p***	***f2***
(Intercept)	−7.281	−10.243 to −4.318	**<0.001**		−0.525	−0.793 to −0.256	**<0.001**		−0.137	−1.326 to 1.052	0.822	
Relevance [non-stepping]	6.616	2.943 to 10.290	**<0.001**	0.118	0.313	0.046 to 0.579	**0.022**	0.089	0.053	−0.869 to 0.976	0.910	1.97 × 10^–4^
Step [step 2]	5.273	1.600 to 8.947	**0.005**	0.048	0.280	0.013 to 0.547	**0.040**	0.071	0.366	−0.557 to 1.288	0.437	0.006
Relevance [non-stepping] * Step [step 2]	−5.857	−11.052 to −0.662	**0.027**	0.079								
**Random Effects**												
Residual variance	21.076				0.222				2.660			
Random intercept variance	6.344				0.059				2.419			
ICC	0.231				0.209				0.476			
Observations	48				48				48			
Marginal R^2^/Conditional R^2^	0.205/0.389			0.260	0.138/0.318			0.161	0.007/0.480			0.007

Referent: Stepping, step 1; ICC, Intraclass Correlation Coefficient; f2, Cohen’s f2, indicating effect size. f2 for the model (bottom row) is calculated from the Marginal R2. Significant *p* values (i.e., <0.05) are bolded.

During the obstacle-crossing trials, the step modified the association between Δ percent correct and relevance, indicated by the significant two-way interaction between relevance and step (Table 2). The pairwise comparisons showed that Δ percent correct at the first stepping location significantly differed compared to the second stepping location [estimated difference: 5.273 (95% CI: 1.31 9.23), *t*_(39.3)_ = 2.694, *p* = 0.010, *d* = 1.149] and the non-stepping locations in step 1 [estimated difference: 6.616 (95% CI: 2.66 10.57), *t*_(39.3)_ = 3.380, *p* = 0.002, *d* = 1.441] and step 2 [estimated difference: 6.032 (95% CI: 2.07 9.99), *t*_(39.3)_ = 3.081, *p* = 0.004, *d* = 1.314]. There was no difference in Δ percent correct between the second stepping location and non-stepping locations (Figure 5D). Our model for Δ percent correct showed an *f*^2^ of 0.260 (Table 2), indicating that the variance explained by this model corresponds to a medium to large effect.

Modifications in visual performance (Δ percent correct) at the first stepping location may be attributed to adaptations in visual sensitivity (Δ *d’*, Figure 5E) rather than response bias (Δ β, Figure 5F). For visual sensitivity, the analysis showed that participants had decreased Δ *d’* at stepping locations compared to non-stepping locations (*p* = 0.022) and decreased Δ *d’* in the first step compared to the second step (*p* = 0.040), but there was no two-way interaction (Figure 5E; Table 2). Our model for Δ *d’* showed an *f*^2^ of 0.160 (Table 2), indicating that the variance explained by this model corresponds to a medium effect. For response bias, there were no associations in Δ β with relevance or step (model *f*^2^ = 0.007, Figure 5F; Table 2).

## Discussion

The limitation of central (foveal) vision is attending to only one area of interest (Carrasco et al., 2001), making peripheral vision crucial for covertly attending to multiple relevant locations (Baldauf et al., 2006) and maintaining a body representation relative to areas of interest (Rietdyk and Rhea, 2006). Visualize an obstacle in your path; we do not directly gaze at our feet as we approach and step over the obstacle. Instead, we select future stepping locations using central vision and presumably use peripheral vision for planning one or more steps ahead during obstacle crossing. To probe the role of peripheral visual input in skilled walking, we developed a new protocol to test the distribution of sustained covert visual attention for planning foot placement during obstacle-crossing planning. The results of this study support the use of psychophysical approaches to track the modulation of peripheral visual attention during a walking task in humans. Our results suggest that initiating a step to begin obstacle crossing decreases visual processing at the first of two stepping locations and compared to remaining stationary. This may indicate that the brain uses peripheral vision to plan one step at a time during obstacle crossing, contrasting the brain’s use of central vision for planning two or more steps in advance.

We believe that we captured changes in covert visual attention during the planning phase of obstacle crossing. We accomplished this by restricting step onset times, obliging participants to initiate obstacle crossing quickly. On average, participants took 741 ms to initiate stepping, which is well after the presentation of the Gabor patch (which was presented 300 ms after the cue for a duration of 77.8 ms). Our estimation of step onset time is consistent with other biological markers of step onset in the literature. Previous studies, using force plate data, report step onsets of between 650 and 710 ms (these experiments also used an arrow cue to indicate to their participants which foot to step with first; Uemura et al., 2013a,b; Braquet et al., 2020). The step onset times from the literature are associated with anticipatory postural adjustments and cortical activity over the parietal and occipital lobes that begin ~200–350 ms (Uemura et al., 2013a,b; Braquet et al., 2020) and ~100–450 ms (Braquet et al., 2020), respectively, after the onset of a movement cue; these early postural and cortical changes are indicators of movement planning (Braquet et al., 2020). The overlap between step onset times in our study with values from the literature supports our assertion that with this protocol, we were able to prompt our participants to begin preparing to step after the movement cue and, therefore, visually discriminate the Gabor patch during the planning phase of the movement.

Importantly, the Gabor patch appeared with equal probability at one of the four potential stepping locations irrespective of whether the cue appeared as a horizontal bar or a right/left arrow. This makes it unlikely that the shifts in attention observed in our study were based on Gabor patch expectancy. In addition, the *d’* results indicate that the decline in visual performance during obstacle-crossing planning likely relates to a decrease in visual sensitivity rather than a general bias for responding “right” or “left” to the orientation of the Gabor patch.

Reaching studies have shown that preparing a reach results in enhanced visual performance at the movement location at short cue-test intervals (~100 ms; Baldauf et al., 2006; Baldauf, 2011; Rolfs et al., 2013; Stewart et al., 2019; Mahon et al., 2020), followed by a decrease in performance at the movement location at longer cue-test intervals (>250–300 ms; Deubel and Schneider, 2003; Stewart et al., 2019). For this first iteration of the protocol, we tested visual performance and sensitivity using a relatively long cue-test interval of 300 ms. At such longer cue-test intervals, we anticipated a reduction in visual performance and sensitivity to movement-relevant locations, indicating a re-direction of attention and implying that movement planning is complete, as per the concept of inhibition of return—once the cued location is sufficiently processed, attention is inhibited at the cued location and re-directed to other “novel” task-relevant (uncued) locations (Posner, 1980; Posner and Cohen, 1984; Posner et al., 1985). Thus, based on reaching studies (Deubel and Schneider, 2003; Baldauf et al., 2006; Baldauf, 2011; Rolfs et al., 2013; Stewart et al., 2019; Mahon et al., 2020), we could assume that planning the first step led to a transient and reflexive enhancement of attention to the first stepping location, followed by the inhibition of attention at this location once it was processed and the movement plan for the first step formed. Future studies using this protocol with shorter cue-test intervals would be required to investigate whether the time course of modulation of visual attention during skilled walking generalizes from that expected from our understanding of the control of upper limb tasks.

The different task-relevance and functions of the first and second steps, which participants presumably implicitly know, may also explain the dissimilarity of covert attention directed to these locations. The first step positions the foot in front of the obstacle at an appropriate distance to ensure clearance over the obstacle as the leading leg (first leg to step over obstacle). The second step positions the opposite foot at an appropriate distance from the obstacle to become the support leg as the leading leg steps over the obstacle; this leg then steps over the obstacle as the trailing leg (second leg to step over obstacle). Furthermore, previous studies have suggested that the lead and trail limbs are controlled independently when obstacle crossing. For example, blocking lower peripheral visual input during obstacle crossing (Mohagheghi et al., 2004), adding a mass to the lead limb (Lajoie et al., 2012), or passively controlling the lead limb over the obstacle (Lajoie et al., 2012) does not modify trail limb clearance. Given that the placement of the first step is more critical for avoiding obstacle contact than the second step (Patla and Greig, 2006), visual performance at the first stepping location may have been modified by dual-task interference between attentional networks for action and perception. Studies investigating dual-task action-perception interference have reported a decline in perceptual performance (Liu et al., 2008; Göhringer et al., 2018) alongside changes in action planning (Liu et al., 2008; Mahon et al., 2020) and execution (Göhringer et al., 2018; Mahon et al., 2020) (i.e., increased reaction time and decreased end-point accuracy). In our study, we observed a decline in perceptual performance at the first stepping location; however, the perceptual task did not influence step onset times (reaction time measure) nor significantly alter foot placement. We did not ask participants to step on a target accurately, which could explain a lack of interference effect on foot placement. However, if the perceptual task interfered with action planning, we would have expected the placement of the Gabor patch at the first stepping location to influence the step onset time to this location. Instead, we observed consistent step onset times irrespective of Gabor patch placement. Thus, we favor an inhibition of the return mechanism rather than interference at this time.

Visual performance at the second stepping and non-stepping locations remained close to 90% irrespective of whether the participant had to remain stationary or initiate stepping. It is possible that these areas were selected for perception, even though they were not selected for action. Because each location was a potential Gabor patch site, the brain may have deployed sustained visual attention to both the second stepping and non-stepping locations. Moreover, during stationary trials, visual performance did not differ across the four locations, suggesting that the brain placed equal priority on information processing at all locations relevant to the task. This interpretation is also supported by a recent reaching study that showed that letter discrimination is enhanced at both reaching and non-reaching locations with movement-relevant feedback (Mahon et al., 2020).

The pace at which participants completed the trials may have been another factor influencing how visual attention was distributed across stepping locations during obstacle crossing. Reaching studies have shown that changing the timing of sequential reaches (i.e., fast vs. slow) alters visual performance at movement locations (Baldauf, 2011), indicating that visual attention is selectively deployed based on the speed of sequential movements. In our study, participants took two steps and stepped over an obstacle at their self-selected pace. The distance between stepping locations was ~56 cm, and the interval between left and right steps during regular overground walking is typically 500–600 ms (Matthis et al., 2018). The obstacle was always in view, so planning for the second stepping location could have occurred any time after that for the first stepping location.

There are several possible extensions of this paradigm for future research. To further probe the time course of the deployment of covert visual attention, researchers could test a range of short and long cue-test intervals beyond that used in the present study, as we discussed above. In addition, to compel a wider distribution of visual attention over multiple stepping locations, researchers could present the obstacle position for a limited time at the beginning of each trial or remove the obstacle altogether and use protocols like others (Reynolds and Day, 2005; Matthis et al., 2017; Domínguez-Zamora et al., 2018) where participants need to step accurately onto specific targets on the floor. Another extension could involve introducing a secondary task to simulate real-world situations, like talking while walking or having to locate a street sign or store, which would further tax the attentional system. Future iterations of this protocol could also manipulate the time between steps, which can be achieved by verbally instructing participants to take steps at a fast or slow speed and quantified by foot kinematics, to probe the limits of visual attention as movement speed increases. Terrain, age, and neurological injury could differentially affect these limits and shed useful insights on the factors predicting the attentional capacity of walking.

Overall, we introduce a new approach, with many possible extensions, to identify the time course of visual attention and the role of peripheral vision during obstacle crossing. In the future, this paradigm may help identify the effects of aging or neurological injury on movement planning to inform rehabilitation strategies to enhance community mobility. The results of this study suggest that sustained covert visual attention is only reduced at the first stepping location during obstacle-crossing planning. Whereas the results of previous studies have indicated that humans use central vision to plan two steps ahead during obstacle crossing, our results here suggest the deployment of covert attention for preparing for the imminent step. Since the brain uses peripheral vision to facilitate a variety of goal-directed motor behaviors, our results may generalize to other everyday walking situations, such as walking across different terrain or where precise foot placement is required, in addition to stepping over obstacles.

## Data Availability Statement

The original contributions presented in the study are included in the article, further inquiries can be directed to the corresponding author.

## Ethics Statement

The studies involving human participants were reviewed and approved by University of British Columbia Clinical Research Ethics Board. All participants provided their written informed consent to participate in this study.

## Author Contributions

RM, DM, and TL conceptualized and designed the research. RM and MC performed the experiments and primary analysis. RM prepared the figures. RM, DM, and TL interpreted the results, and drafted and revised the manuscript. All authors contributed to the article and approved the submitted version.

## References

[B1] Amitay S. Irwin A. Hawkey D. J. C. Cowan J. A. Moore D. R. (2006). A comparison of adaptive procedures for rapid and reliable threshold assessment and training in naive listeners. J. Acoust. Soc. Am. 119, 1616–1625. 10.1121/1.2164988 16583906

[B2] Baldauf D. (2011). Chunking movements into sequence: the visual pre-selection of subsequent goals. Neuropsychologia 49, 1383–1387. 10.1016/j.neuropsychologia.2011.01.041 21295048

[B3] Baldauf D. Wolf M. Deubel H. (2006). Deployment of visual attention before sequences of goal-directed hand movements. Vis. Res. 46, 4355–4374. 10.1016/j.visres.2006.08.021 17034829

[B4] Bates D. Mächler M. Bolker B. Walker S. (2015). Fitting linear mixed-effects models using lme4. J. Stat. Softw. 67, 1–48. 10.18637/jss.v067.i01

[B6] Bisley J. W. Goldberg M. E. (2010). Attention, intention and priority in the parietal lobe. Annu. Rev. Neurosci. 33, 1–21. 10.1146/annurev-neuro-060909-152823 20192813 PMC3683564

[B7] Braquet A. Bayot M. Tard C. Defebvre L. Derambure P. Dujardin K.. (2020). A new paradigm to study the influence of attentional load on cortical activity for motor preparation of step initiation. Exp. Brain Res. 238, 643–656. 10.1007/s00221-020-05739-5 32025766

[B8] Carrasco M. (2011). Visual attention: the past 25 years. Vis. Res. 51, 1484–1525. 10.1016/j.visres.2011.04.012 21549742 PMC3390154

[B9] Carrasco M. Talgar C. P. Cameron E. L. (2001). Characterizing visual performance fields: effects of transient covert attention, spatial frequency, eccentricity, task and set size. Spat. Vis. 15, 61–75. 10.1163/15685680152692015 11893125 PMC4332623

[B10] Cohen J. (1992). A power primer. Psychol. Bull. 112, 155–159. 10.1037//0033-2909.112.1.155 19565683

[B11] Costa R. Q. M. da Pompeu J. E. Mello D. D. de Moretto E. Rodrigues F. Z. Santos M. D. D.. (2018). Two new virtual reality tasks for the assessment of spatial orientation Preliminary results of tolerability, sense of presence and usability. Dement. Neuropsychol. 12, 196–204. 10.1590/1980-57642018dn12-020013 29988338 PMC6022991

[B12] Deubel H. Schneider W. X. (2003). Delayed saccades, but not delayed manual aiming movements, require visual attention shifts. Ann. N Y Acad. Sci. 1004, 289–296. 10.1196/annals.1303.026 14662468

[B13] Domínguez-Zamora F. J. Gunn S. M. Marigold D. S. (2018). Adaptive gaze strategies to reduce environmental uncertainty during a sequential visuomotor behaviour. Sci. Rep. 8:14112. 10.1038/s41598-018-32504-0 30237587 PMC6148321

[B14] Drew T. Marigold D. S. (2015). Taking the next step: cortical contributions to the control of locomotion. Curr. Opin. Neurobiol. 33, 25–33. 10.1016/j.conb.2015.01.011 25643847

[B15] Fecteau J. H. Munoz D. P. (2006). Salience, relevance and firing: a priority map for target selection. Trends Cogn. Sci. 10, 382–390. 10.1016/j.tics.2006.06.011 16843702

[B16] Göhringer F. Löhr-Limpens M. Schenk T. (2018). The visual guidance of action is not insulated from cognitive interference: a multitasking study on obstacle-avoidance and bisection. Conscious. Cogn. 64, 72–83. 10.1016/j.concog.2018.07.007 30093260

[B17] Gottlieb J. (2007). From thought to action: the parietal cortex as a bridge between perception, action and cognition. Neuron 53, 9–16. 10.1016/j.neuron.2006.12.009 17196526

[B18] Grier R. A. (2015). How high is high? A meta-analysis of NASA-TLX global workload scores. Proc. Hum. Factors Ergon. Soc. Annu. Meet. 59, 1727–1731. 10.1177/1541931215591373

[B19] Grubb M. A. White A. L. Heeger D. J. Carrasco M. (2015). Interactions between voluntary and involuntary attention modulate the quality and temporal dynamics of visual processing. Psychon. Bull. Rev. 22, 437–444. 10.3758/s13423-014-0698-y 25117089 PMC4326639

[B20] Handy T. C. Soltani M. Mangun G. R. (2001). Perceptual load and visuocortical processing: event-related potentials reveal sensory-level selection. Psychol. Sci. 12, 213–218. 10.1111/1467-9280.00338 11437303

[B21] Hart S. G. Staveland L. E. (1988). “Development of NASA-TLX (Task Load Index): results of empirical and theoretical research,” in Human Mental Workload, eds Hancock P. A. Meshkati N. (North-Holland: Elsevier), 139–183. 10.1016/s0166-4115(08)62386-9

[B22] Hartig F. (2020). DHARMa: residual diagnostics for hierarchical (multi-level/mixed) regression models. Available online at: https://cran.r-project.org/web/packages/DHARMa/vignettes/DHARMa.html.

[B23] Hautus M. J. (1995). Corrections for extreme proportions and their biasing effects on estimated values of d^′^. Behav. Res. Methods Instrumen. Comput. 27, 46–51. 10.3758/BF03203619

[B24] Hoffman L. (2015). Longitudinal Analysis: Modeling Within-Person Fluctuation and Change. New York: Routledge.

[B25] Lajoie K. Bloomfield L. W. Nelson F. J. Suh J. J. Marigold D. S. (2012). The contribution of vision, proprioception and efference copy in storing a neural representation for guiding trail leg trajectory over an obstacle. J. Neurophysiol. 107, 2283–2293. 10.1152/jn.00756.2011 22298832

[B26] Leek M. R. (2001). Adaptive procedures in psychophysical research. Percept. Psychophys. 63, 1279–1292. 10.3758/bf03194543 11800457

[B27] Lenth R. V. (2020). emmeans: estimated marginal means, aka least-squares means. Available online at: https://github.com/rvlenth/emmeans.

[B28] Levitt H. (1971). Transformed up-down methods in psychoacoustics. J. Acoust. Soc. Am. 49, 467–477. 10.1121/1.1912375 5541744

[B29] Ling S. Carrasco M. (2006). Sustained and transient covert attention enhance the signal via different contrast response functions. Vis. Res. 46, 1210–1220. 10.1016/j.visres.2005.05.008 16005931 PMC1557421

[B30] Liu G. Chua R. Enns J. T. (2008). Attention for perception and action: task interference for action planning, but not for online control. Exp. Brain Res. 185, 709–717. 10.1007/s00221-007-1196-5 18008067

[B31] Lorah J. (2018). Effect size measures for multilevel models: definition, interpretation and TIMSS example. Large-scale Assess. Educ. 6:8. 10.1186/s40536-018-0061-2

[B32] Lüdecke D. (2020). sjPlot: data visualization for statistics in social science. Available online at: https://strengejacke.github.io/sjPlot/.

[B33] Macmillan N. A. Creelman C. D. (2004). Detection Theory. New York: Psychology Press.

[B34] Macmillan N. A. Kaplan H. L. (1985). Detection theory analysis of group data: estimating sensitivity from average hit and false-alarm rates. Psychol. Bull. 98, 185–199. 4034817

[B35] Mahon A. Bendźiūtė S. Hesse C. Hunt A. R. (2020). Shared attention for action selection and action monitoring in goal-directed reaching. Psychol. Res. 84, 313–326. 10.1007/s00426-018-1064-x 30097712 PMC7040085

[B36] Makowski D. (2018). The psycho package: an efficient and publishing-oriented workflow for psychological science. J. Open Source Softw. 3:470. 10.21105/joss.00470

[B37] Marigold D. S. (2008). Role of peripheral visual cues in online visual guidance of locomotion. Exerc. Sport Sci. Rev. 36, 145–151. 10.1097/JES.0b013e31817bff72 18580295

[B38] Marigold D. S. Patla A. E. (2007). Gaze fixation patterns for negotiating complex ground terrain. Neuroscience 144, 302–313. 10.1016/j.neuroscience.2006.09.006 17055177

[B39] Matthis J. S. Barton S. L. Fajen B. R. (2017). The critical phase for visual control of human walking over complex terrain. Proc. Natl. Acad. Sci. U S A 144, E6720–E6729. 10.1073/pnas.1611699114 PMC5558990 28739912

[B40] Matthis J. S. Yates J. L. Hayhoe M. M. (2018). Gaze and the control of foot placement when walking in natural terrain. Curr. Biol. 28, 1224–1233.e5. 10.1016/j.cub.2018.03.008 29657116 PMC5937949

[B41] Mohagheghi A. A. Moraes R. Patla A. E. (2004). The effects of distant and on-line visual information on the control of approach phase and step over an obstacle during locomotion. Exp. Brain Res. 155, 459–468. 10.1007/s00221-003-1751-7 14770275

[B42] Müller H. J. Rabbitt P. M. (1989). Reflexive and voluntary orienting of visual attention: time course of activation and resistance to interruption. J. Exp. Psychol. Hum. Percept. Perform. 15, 315–330. 10.1037//0096-1523.15.2.315 2525601

[B43] Musselman K. E. Yang J. F. (2007). Walking tasks encountered by urban-dwelling adults and persons with incomplete spinal cord injuries. J. Rehabil. Med. 39, 567–574. 10.2340/16501977-0090 17724557

[B500] Mutasim A. K. Stuerzlinger W. Batmaz A. U. (2020). “Gaze tracking for eye-hand coordination training systems in virtual reality,” in Extended Abstracts of the 2020 CHI Conference on Human Factors in Computing Systems, (Honolulu, HI, United States), 1–9. 10.1145/3334480.3382924

[B44] Nakayama K. Mackeben M. (1989). Sustained and transient components of focal visual attention. Vis. Res. 29, 1631–1647. 10.1016/0042-6989(89)90144-2 2635486

[B45] Niang A. E. S. McFadyen B. J. (2004). Adaptations in bilateral mechanical power patterns during obstacle avoidance reveal distinct control strategies for limb elevation versus limb progression. Motor Control 8, 160–173. 10.1123/mcj.8.2.160 15118200

[B46] Pallier C. (2002). Computing discriminability and bias with the R software. Available online at: https://www.pallier.org/pdfs/aprime.tif.

[B47] Pastel S. Chen C.-H. Petri K. Witte K. (2020). Effects of body visualization on performance in head-mounted display virtual reality. PLoS One 15:e0239226. 10.1371/journal.pone.0239226 32956420 PMC7505416

[B48] Patla A. E. (1998). How is human gait controlled by vision. Ecol. Psychol. 10, 287–302. 10.1080/10407413.1998.9652686

[B49] Patla A. E. Greig M. (2006). Any way you look at it, successful obstacle negotiation needs visually guided on-line foot placement regulation during the approach phase. Neurosci. Lett. 397, 110–114. 10.1016/j.neulet.2005.12.016 16413969

[B50] Patla A. E. Vickers J. N. (1997). Where and when do we look as we approach and step over an obstacle in the travel path? Neuroreport 8, 3661–3665. 10.1097/00001756-199712010-00002 9427347

[B52] Piumsomboon T. Lee G. Lindeman R. W. Billinghurst M. (2017). “Exploring natural eye-gaze-based interaction for immersive virtual reality,” in 2017 IEEE Symposium on 3D User Interfaces (3DUI), (Los Angeles, CA, USA), 36–39. 10.1109/3dui.2017.7893315

[B53] Posner M. I. (1980). Orienting of attention. Q. J. Exp. Psychol. 32, 3–25. 10.1080/00335558008248231 7367577

[B54] Posner M. I. Cohen Y. (1984). Components of visual orienting of attention. Attent. Perform. 32, 531–556.

[B55] Posner M. I. Rafal R. D. Choate L. S. Vaughan J. (1985). Inhibition of return: neural basis and function. Cogn. Neuropsychol. 2, 211–228. 10.1080/02643298508252866

[B320] R Core Team (2020). R: A language and environment for statistical computing. Vienna, Austria: R Foundation for Statistical Computing. Available online at: https://www.R-project.org/.

[B56] Reynolds R. F. Day B. L. (2005). Visual guidance of the human foot during a step. J. Physiol. 569, 677–684. 10.1113/jphysiol.2005.095869 16179363 PMC1464243

[B57] Rietdyk S. Rhea C. K. (2006). Control of adaptive locomotion: effect of visual obstruction and visual cues in the environment. Exp. Brain Res. 169, 272–278. 10.1007/s00221-005-0345-y 16421728

[B58] Rolfs M. Carrasco M. (2012). Rapid simultaneous enhancement of visual sensitivity and perceived contrast during saccade preparation. J. Neurosci. 32, 13744–13752a. 10.1523/JNEUROSCI.2676-12.2012 23035086 PMC3498617

[B59] Rolfs M. Lawrence B. M. Carrasco M. (2013). Reach preparation enhances visual performance and appearance. Philos. Trans. R. Soc. Lond. B Biol. Sci. 368:20130057. 10.1098/rstb.2013.0057 24018719 PMC3758200

[B60] Siedlecka M. Wereszczyński M. Paulewicz B. Wierzchoń M. (2020). Visual awareness judgments are sensitive to accuracy feedback in stimulus discrimination tasks. Conscious. Cogn. 86:103035. 10.1016/j.concog.2020.103035 33157486

[B61] Stanislaw H. Todorov N. (1999). Calculation of signal detection theory measures. Behav. Res. Methods Instrum. Comput. 31, 137–149. 10.3758/bf03207704 10495845

[B62] Stewart E. E. M. Verghese P. Ma-Wyatt A. (2019). The spatial and temporal properties of attentional selectivity for saccades and reaches. J. Vis. 19:12. 10.1167/19.9.12 31434108 PMC6707227

[B63] Uemura K. Oya T. Uchiyama Y. (2013a). Effects of speed and accuracy strategy on choice step execution in response to the flanker interference task. Hum. Mov. Sci. 32, 1393–1403. 10.1016/j.humov.2013.07.007 24060225

[B64] Uemura K. Oya T. Uchiyama Y. (2013b). Effects of visual interference on initial motor program errors and execution times in the choice step reaction. Gait Posture 38, 68–72. 10.1016/j.gaitpost.2012.10.016 23195857

[B66] Witmer B. G. Jerome C. J. Sunger M. J. (2005). The factor structure of the presence questionnaire. Presence: Teleoperators and Virtual Environments 14, 298–312. 10.1162/105474605323384654

[B65] Witmer B. G. Singer M. J. (1998). Measuring presence in virtual environments: a presence questionnaire. Presence: Teleoperators and Virtual Environments 7, 225–240. 10.1162/105474698565686

